# Effects of psychological resilience on social media information-sharing behavior in older adults: mediating role of technology anxiety and perceived enjoyment

**DOI:** 10.3389/fpsyg.2025.1595688

**Published:** 2025-09-05

**Authors:** Jing An, Ziyue Xiang, Kexin Wan, Yujie Yang, Xuanyu Zhu, Jinlong An

**Affiliations:** ^1^School of Management, Nanjing University of Posts and Telecommunications, Nanjing, Jiangsu, China; ^2^The Fourth People’s Hospital of Shenzhen, Shenzhen, Guangdong, China; ^3^First People’s Hospital of Changshu City, Hospital Affiliated to Soochow University, Changshu, Jiangsu, China

**Keywords:** older adults, psychological resilience, information sharing behavior, technology anxiety, uses and gratification theory

## Abstract

**Background:**

Against the backdrop of the rapid development of information technology, accelerated population aging, and the prominent “digital divide” among older adults, the information-sharing behavior of older adults on social media has attracted significant attention. Understanding the mechanism behind their information-sharing on social media is crucial for bridging the digital divide and promoting healthy aging.

**Methods:**

This study combines Technology Acceptance Model and Uses and Gratification Theory to construct a theoretical model with psychological resilience as the core, and explores its influence mechanism with technology anxiety, social interaction, perceived enjoyment, and perceived ease of use on the information sharing behavior of older adults. A questionnaire survey was conducted among Chinese older adults aged 55 and above, from which a total of 297 valid responses were collected. Structural Equation Modeling (SEM) was then used to conduct an empirical analysis.

**Results:**

The results show that psychological resilience, social interaction, perceived enjoyment, and perceived ease of use significantly positively affect the information sharing behavior of older adults, while technology anxiety negatively inhibits information sharing and significantly reduces the perceived ease of use of social media of older adults. Psychological resilience directly influences information sharing behavior and indirectly promotes information sharing behavior by reducing technology anxiety, but it has no significant direct correlation with perceived enjoyment. Social interaction indirectly promotes information sharing through the mediation of perceived enjoyment.

**Discussion:**

This study not only provides a reference for understanding the information-sharing behavior of older adults, promoting their integration into the digital society, and facilitating the harmonious coexistence of digitalization and aging, but also offers deeper insights into how to support older adults in overcoming digital barriers and improving their quality of life through meaningful online interactions.

## 1 Introduction

The rapid advancement of information technology has profoundly transformed everyday life, particularly in how individuals communicate and access information. Social media platforms such as WeChat, TikTok, Weibo, and REDnote have emerged as central tools for information exchange due to their openness, interactivity, and immediacy. Among the many forms of digital interaction, information sharing has become a key activity that shapes social experiences across age groups. In parallel, China is undergoing a significant demographic shift. By the end of 2024, the population aged 60 and over reached 310.31 million, accounting for 22.0% of the total population—an increase of more than 13 million in just 1 year. This rapid aging trend presents both social and technological challenges. At the same time, the elderly population is increasingly participating in the digital world. According to the “54th Statistical Report on Internet Development in China” released by the China Internet Network Information Center (CNNIC), the internet penetration rate has risen to 78%, with individuals aged 60 and above representing 14.3% of total users. Despite this growth, older adults remain a vulnerable group in the digital environment due to age-related cognitive and sensory decline (Ghasemaghaei et al., 2019; Tams, 2022). This can lead to difficulties in technology adoption and effective information use (Cheng et al., 2023). As digital integration becomes essential for daily life, understanding how older adults engage in information sharing on social media is a growing area of interest. Specifically, identifying the psychological and technological barriers they face is critical for fostering digital inclusion and promoting active and healthy aging.

Older adults have maintained prominent attention as vital structural components of society. Bridging the digital divide to facilitate older adults’ integration into digital society and safeguard their digital entitlements has emerged as a critical research imperative. Achieving these objectives is fundamental to enhancing older adults’ quality of life and well-being, while concurrently advancing active and healthy aging paradigms. Within this context, information sharing behaviors of older adults in social media environments have gained significant scholarly attention in the field of information behavior studies. Entering the elderly stage, individuals’ psychological state gradually changes, such as stronger demand for emotions and more peaceful attitude toward life. These psychological changes make them pay more attention to emotional communication and experience inheritance when sharing information. At the same time, the evolution of life demands from material concerns toward spiritual fulfillment has elevated the importance of health information and social interaction (Kim et al., 2019). This shift subsequently manifests in distinctive information sharing behavioral patterns. For example, older adults are more inclined to share health knowledge, family chores and other content closely related to life with family or friends, so as to improve social life and reduce loneliness (Ramírez-Correa et al., 2019), thus improving life satisfaction (Zhou, 2018).

While technological advancements provide diverse information-sharing channels (e.g., social media, mobile apps) for older adults, technological complexity generates technical pressure. Consequently, negative attitudes emerge among this population (Cheng et al., 2023), manifested as technology anxiety (Chew et al., 2023) and information avoidance (Golman et al., 2017; Gu et al., 2025), which hinder their ability to fully leverage these tools for information sharing. In addition, the frequency and quality of social interactions (Jo and Baek, 2023; Rauwers et al., 2019), perceived enjoyment from mobile applications (Yang and Lin, 2019), perceived ease of use (Sarmiento et al., 2024) and other factors are subtly affecting the information sharing behaviors of older adults. However, at present, there is still a lack of systematic and in-depth exploration on the mechanism of these influencing factors and their relationship. Looking back at past studies, some progress has been made in the discussion of the information sharing behavior of older adults, and it is clear that various factors such as technology anxiety, social interaction, and perceived enjoyment have an impact on them. However, psychological resilience, as an important index to measure individuals’ resilience in the face of stress and adversity, has been neglected in the research of information sharing behaviors of older adults. Older adults with higher psychological resilience may be better at dealing with the problems encountered in the process of information sharing, such as the interference of online rumors and the difficulty of technical operation, so as to participate in information sharing more actively. Secondly, the comprehensive mechanism of technology anxiety, social interaction, perceived enjoyment and perceived ease of use in information sharing behavior is not yet clear. These factors do not exist in isolation, but are intertwined and influence each other, and jointly act on the information sharing decision of older adults. Consequently, based on the fact that the aging of Chinese social population is deepening and the digital divide among older adults, this study will conduct empirical research by constructing an information sharing model for older adults under the background of social media.

This study aims to explore the factors influencing older adults’ information-sharing behavior on social media in the context of China’s aging society. By integrating the Technology Acceptance Model (TAM) and the Uses and Gratification Theory (UGT), and incorporating variables such as psychological resilience, perceived enjoyment, social interaction, technology anxiety, and perceived ease of use, this research seeks to develop a comprehensive model that reveals the mechanisms driving older adults’ digital engagement. The study also investigates the mediating roles of perceived enjoyment and technology anxiety, contributing to a deeper understanding of how to support elderly individuals in overcoming digital barriers and enhancing their quality of life through meaningful online interaction.

## 2 Theoretical basis and research hypothesis

### 2.1 Theoretical basis

#### 2.1.1 Uses and gratification theory

Uses and Gratification Theory (UGT) is a theoretical framework that explains why and how individuals actively seek specific media to meet their specific needs (Severin and Tankard, 2001). UGT emphasizes that users consciously choose media to meet their needs, which cover multiple dimensions such as social interaction, entertainment, relaxation, etc. (Whiting and Williams, 2013), and is widely used in the field of communication technology of new media (Pillai et al., 2025). UGT reveals the impact of convenience and entertainment on the intention to adopt information and communication technologies, while noting that the state of information seeking and sharing experiences enhances users’ intention to continue using the technology (Gallego et al., 2016). Scholars have conducted extensive research on the uses and gratification of online social media platforms, covering multiple dimensions such as experiential happiness, entertainment and relaxation, social interaction, emotional support, convenience, information sharing and information seeking (Kaur et al., 2020), these needs are particularly prominent in older adults, as they may face issues such as social isolation, limited access to information, etc. Studies have shown that hedonic factors (entertainment) and socialization are the main driving forces for older adults to participate in social networking sites (Kim et al., 2019). Hedonic motivation refers to sharing media content to entertain oneself, satisfy happiness needs, release emotions, relieve anxiety and escape stress (Sun and Xie, 2024; Thompson et al., 2020), and hedonism is positively correlated with people’s intention to share information on social media (Hur et al., 2017). In addition, social networking also plays an important role in the sharing of information on social media. Social motivation motivates people to share personal experiences, knowledge, and resources on social media (Oh and Syn, 2015). Social media provides a convenient alternative channel for older adults who may struggle to maintain face-to-face interaction with family and friends due to physical restrictions or geographical distancing as they age. By sharing information, older adults are able to not only stay connected with others, but also receive entertainment and emotional support, thus alleviating loneliness. Taken together, UGT provides a comprehensive theoretical framework for understanding social media information sharing behavior of older adults. By analyzing social interaction and entertainment needs (perceived enjoyment), we can gain a deeper understanding of older adults’ information sharing behavior on social media.

#### 2.1.2 Technology acceptance model

Technology Acceptance Model (TAM) is derived from Theory of Reasoned Action (TRA) proposed by Fishbeinand Ajzen (Fishbein and Ajzen, 2011). Davis revised the TRA in 1989 and proposed the TAM model when using rational behavior theory to study users’ acceptance of information systems (Davis, 1985). TAM believes that individuals’ intentions and actions to adopt new technologies depend on perceived usefulness and perceived ease of use (Duong et al., 2023). Among them, perceived ease of use is defined as a user’s perception of the level of complexity in using a particular technology (Davis and Venkatesh, 1996), and individuals tend to show a high willingness to adopt a new technology when they feel that they can learn how to use it quickly and easily (Del Giudice et al., 2023). In the context of social media, the information sharing behaviors of older adults is also influenced by perceived ease of use. Perceived ease of use reflects older adults’ perception of the ease of information sharing on social media platforms, and lower perceived ease of use may increase technology anxiety, thus inhibiting their information sharing intention and behavior. With the continuous deepening of research, researchers have gradually integrated multiple factors such as perceived enjoyment, perceived risk (Driediger and Bhatiasevi, 2019), social pressure (Yap et al., 2022), subjective norms (Kim and Han, 2022), and technology anxiety (Rajak and Shaw, 2021) into TAM, deriving extended models such as TAM2 (Venkatesh and Davis, 2000) and UTAUT (Venkatesh et al., 2003). For example, Hoque and Sorwar conducted empirical research based on UTAUT and found that effort expectation, social influence and technology anxiety had a greater impact on the intention of older adults to use mobile health services (Hoque and Sorwar, 2017). In the context of social media, the information sharing behaviors of older adults is also affected by technology anxiety. Technology anxiety as a negative factor may hinder the intention and behavior of information sharing in older adults, especially if technology operations are complex or lack of support.

### 2.2 Research hypothesis

#### 2.2.1 Psychological resilience

Psychological resilience is often used in psychological and sociological research and is usually defined as people’s ability to actively adapt to adversity (Fletcher and Sarkar, 2013; Sisto et al., 2019). In general, individuals with high levels of psychological resilience exhibit high levels of energy and optimism (Block and Block, 2014). On the contrary, people with low psychological resilience often have negative emotions and behaviors when faced with difficulties. Today, with the rapid development of social media and information technology, the psychological resilience of older adults will affect their mood and ability to adapt to new technologies. Optimistic older adults can often actively seek Internet knowledge and skills to adapt to the new environment. Studies on information systems suggest that consumer resilience may play an important role in alleviating technological stress (Bermes, 2021). However, high levels of psychological resilience may be associated with low depression and low anxiety (Wang et al., 2023), and the higher the psychological resilience, the lower the incidence of anxiety (Scali et al., 2012). Therefore, it can be inferred that older adults with low psychological resilience are prone to negative emotions such as anxiety when seeking and sharing knowledge and skills on social media. Based on this, we put forward the following hypotheses:

*H1*: Older adults with low level of psychological resilience are prone to technology anxiety.

In terms of the results produced by resilience, resilience leads to greater well-being (Troy et al., 2023). Psychological resilience is closely linked to the formation and maintenance of positive emotions and optimistic attitudes in individuals (Cuhadar et al., 2016; Sisto et al., 2019). Studies have shown that positive emotions create a positive atmosphere, enhance feelings of pleasure and satisfaction, leading to an increase in intrinsic motivation, and that individuals who experience positive emotions are more likely to get pleasure from activities and are willing to engage in them; On the contrary, negative emotions create a negative atmosphere that diminishes feelings of pleasure and satisfaction, thus reducing intrinsic motivation and motivation to participate in activities (Jo and Baek, 2023). Antonovsky believes that individuals with high psychological resilience can grow in adversity and maintain a state of happiness (Antonovsky, 1987). This view is particularly significant among older adults, which is manifested in the fact that they are more likely to perceive happiness and satisfaction in the process of information sharing, so that they can share information more actively. Within the research field of social media use behavior, Psychological resilience is considered to be negatively associated with problem behaviors such as problematic social networking site use (Hou et al., 2017). Furthermore, there are studies showing that positive/negative emotional arousal is associated with an enhanced propensity to disseminate information (Martel et al., 2020). Alena Bermes’ research further states that consumer psychological resilience plays an important role in resisting the online sharing of false information (Bermes, 2021). To sum up, we put forward the following hypotheses:

*H2*: The psychological resilience of older adults positively affects their perceived enjoyment.

*H3*: The psychological resilience of older adults positively affects their information sharing behavior.

#### 2.2.2 Technology anxiety

Worry and anxiety have become one of the main obstacles to the use of technology by the elderly (Ha and Park, 2020). This anxiety often makes older adults feel nervous when faced with technology use or sharing information, thus weakening their perceived ease of use and information sharing behavior. Antonio’s research on the influence of the elderly’s willingness to use technology further confirmed that technology anxiety has a significant negative impact on perceived ease of use (Martín-García et al., 2022). As the individual’s computer anxiety level increases, its perceived ease of use of specific systems will decrease (Venkatesh, 2000). In the digital age where mobile applications dominate information interaction, technological anxiety as an intermediary mechanism between psychological resilience and information sharing behavior has important research value. Studies have shown that negative emotions can hinder individuals’ concentration on mobile applications by increasing anxiety (Jo and Baek, 2023; Peifer et al., 2014), thereby reducing their willingness to share information. This influence presents differentiated characteristics in the dimension of personality traits: people with neuroticism are significantly more anxious about technology adoption and use (Berner et al., 2023), that is, individuals with high neuroticism, due to their emotional instability, are more prone to triggering negative emotions when faced with technological complexity and uncertainty, leading to heightened technology-related anxiety. It can be seen that psychological resilience plays a key role through dual paths—people with high psychological resilience can effectively buffer the anxiety caused by technical pressure and maintain a positive state of technology use by virtue of their excellent emotional regulation ability, otherwise they are easy to fall into the vortex of anxiety. When technology anxiety continues to accumulate, it will have a multi-dimensional negative impact on information sharing through increased cognitive load and behavioral inhibition mechanisms: excessive worries about operational errors lead to repeated checks to delay sharing timeliness, and information privacy security concerns lead to disengagement behavior (Maduku et al., 2025), thereby terminating sharing behavior. It can be seen that psychological resilience not only directly affects individuals’ adaptability to technical challenges, but also indirectly shapes information sharing behavior patterns by regulating anxiety sensitivity. Based on this, we put forward the following hypotheses:

*H4*: Technology anxiety in older adults negatively affects their perceived ease of use.

*H5*: Technology anxiety in older adults negatively affects their information sharing behavior.

*H6*: Technology anxiety mediates the relationship between psychological resilience and information sharing behavior.

#### 2.2.3 Perceived ease of use

At the moment when the aging society is accelerating, the research on the technology use behavior of older adults has attracted much attention, covering Internet use (Guner and Acarturk, 2020), social network use (Braun, 2013), and the use of remote health monitoring devices (Zhou et al., 2019) and other fields. Perceived ease of use as a key variable in the TAM model is incorporated into the research model in this study. By combing the empirical studies of technology use in the elderly, scholars have found that perceived ease of use (or endeavor expectation) can have a significant impact on various technology adoption (Kavandi and Jaana, 2020). For example, when Filieri et al. used the TAM model to explore the continuous intention of user-generated content (UGC) platform, they found that perceived ease of use positively affects customer satisfaction, thereby promoting the continuous intention of UGC platform (Filieri et al., 2021). For older adults, the convenience of obtaining and sharing information largely affects their enthusiasm for participating in social media activities. When older adults perceive that technology is highly easy to use in the process of information acquisition and sharing, they are more inclined to actively carry out information sharing behaviors. Based on this, this study puts forward the following hypotheses:

*H7*: Perceived ease of use positively affects information sharing behavior in older adults.

#### 2.2.4 Social interaction

UGT suggests that social interaction and fun gratification needs motivate an individual’s ongoing intention to use social networking sites (Chiang, 2013; Kim et al., 2019). This study uses UGT to explain the reasons why older adults make information sharing behaviors, and holds that perceived enjoyment and social interaction are all motivations for older adults to share information, and social gratification increases the sharing behavior of false news (Dabbous and Aoun Barakat, 2023). Studies have proved that groups using Facebook have four needs: “socialization, entertainment, self-state seeking, and information” (Park et al., 2009), and users who share news are motivated to socialize (Lee and Ma, 2012). In the usage context of mobile applications, perceived enjoyment can be enhanced through their communication characteristics, which mainly stems from the fun experience brought by users in the process of interacting with others (Jo and Baek, 2023). Existing research shows that the higher the perceived interactivity, the stronger the sense of enjoyment elicited (Rauwers et al., 2019). Furthermore, Lee found that social interaction on VR devices has a positive impact on perceived enjoyment when studying consumers’ willingness to use virtual reality (VR) devices (Lee et al., 2019). Based on this, we put forward the following hypotheses:

*H8*: Social interaction is positively associated with information sharing behavior in older adults.

*H9*: Social interaction positively affects perceived enjoyment.

#### 2.2.5 Perceived enjoyment

Perceived enjoyment and perceived ease of use will have an impact on the use of social networking sites by older adults (Ramírez-Correa et al., 2019). It is generally believed that users experience more pleasure when using social networking sites as a hedonic system, so their willingness to continue to use the hedonic system will be greater in the future. It can be understood that when people get pleasure and satisfaction from an activity, their likelihood of continuous participation increases (Ryan et al., 2006), older adults are likely to share information more frequently when they get pleasure from sharing it with them. In mobile applications and online social environments, the quality and frequency of social interactions have an important impact on user behavior. Users frequently use mobile applications when they get a sense of pleasure, satisfaction, or accomplishment from their interactions (Jo and Baek, 2023). As a key link in the use of mobile applications, social interaction is directly related to the degree of users’ perceived enjoyment. There are studies showing that online activities are considered more enjoyable when content is assessed as highly interactive, and interactivity is able to have an impact on e-magazine attitudes through perceived enjoyment (Rauwers et al., 2019). This means that in the process of social interaction, rich and high-quality interactive elements, such as instant feedback and in-depth communication, can greatly enhance users’ perception and enjoyment. When users feel this kind of pleasure in social interaction, the pursuit of this positive experience will encourage them to participate more actively in information sharing behavior. For example, on social media platforms, users frequently interact with friends, and get good perceptual enjoyment from comments, likes and other interactions, thus inspiring them to share interesting things, opinions and other information in their lives, so as to maintain and strengthen this positive experience. Based on this, we put forward the following hypotheses:

*H10*: Perceived enjoyment is positively associated with information sharing behavior in older adults.

*H11*: Perceived enjoyment mediates the relationship between social interaction and information sharing behavior.

**Figure 1:**
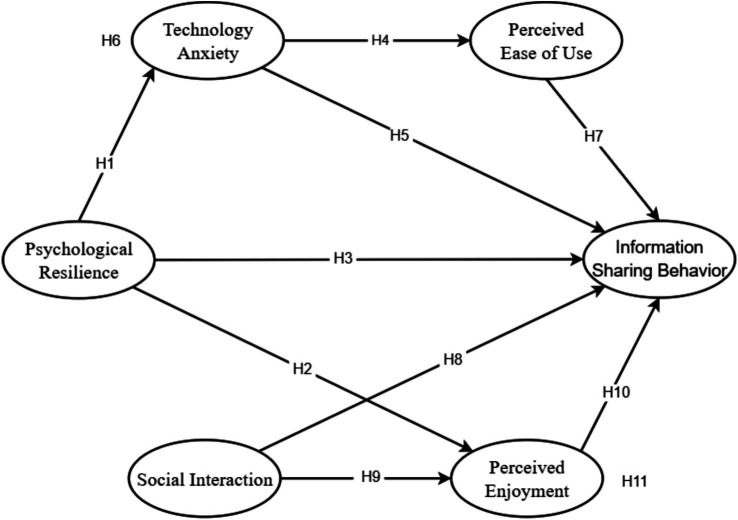
Information sharing behavior model of older adults in the context of social media.

Based on the above hypotheses, we build the following model, as shown in Figure 1.

## 3 Research design

### 3.1 Data collection

This study selected individuals aged 55 and above as research subjects and defined them as older adults. A random sampling method was employed to recruit participants. The questionnaire was collected through the Credamo platform. The questionnaire subjects were older adults who had used social media platforms such as WeChat, Weibo, Tiktok and REDnote. In order to ensure the quality of the questionnaire, it is set that each IP address can only answer once when publishing the questionnaire. The questionnaire was distributed from March, 2023 to June, 2023, which lasted for 4 months, and a total of 313 questionnaires were collected. In this study, abnormal invalid samples such as answering time less than 60 s and choosing the same option for multiple questions in succession were deleted. The final valid samples totaled 297, which met the minimum sample size required by previous studies (Gefen et al., 2000), and the effective rate of sample recovery was 94.9%.

### 3.2 Measure

The data was collected by questionnaire survey. The final questionnaire consists of two parts. The first part is the basic information of the respondent, and the second part is the specific measurement of latent variables, that is, by observing variables. Among them, these measurement items are mainly adapted from existing literature. In this study, we define social media as those online media platforms for people to communicate, post comments, and share knowledge and experience, such as WeChat, Weibo, Tiktok, REDnote, etc. We also pay attention to the exposure time and frequency of social media use by older adults. This study uses the Likert five-point scale to evaluate the observed variables to measure the degree of agreement between the respondents’ own actual situation and the description of the observed variables, graded from “strongly disagree to strongly agree” and balanced in their initial formulation (positive and negative meanings). The specific measurement items and sources are shown in Table 1.

**Table 1:** Measurement items and sources of each latent variable.

Latent variable	Observed variables	Contents	Source
Psychological resilience (PR)	PR1	I think the older I get, the more useless I am	Lou et al. (2023), Wang et al. (2024)
	PR2	I often feel scared or anxious	
	PR3	I often feel lonely	
	PR4	I always look on the bright side of things	
	PR5	I can make my own decisions about my personal affairs	
Technology anxiety (TA)	TA1	Sharing information on social media makes me feel uncomfortable/upset	Wilson et al. (2023)
	TA2	Sharing information on social media makes me nervous	
	TA3	Sharing information on social media does not improve my quality of life	
	TA4	I have doubts about my ability to comment, repost, share information on social media	
Perceived enjoyment (PE)	PE1	Commenting and sharing information on social media makes me fun	Jo and Baek (2023), Ramírez-Correa et al. (2019), Venkatesh (2000)
	PE2	I like to comment, forward and share information in social media such as Weibo, REDnote and WeChat	
	PE3	The process of sharing, reposting and commenting often makes me happy	
Social Interaction (SOI)	SOI1	I will make friends with other people in social media such as Weibo, REDnote and WeChat	An et al. (2020)
	SOI2	By sharing information on social media platforms, I am able to help other users and get help at the same time	
	SOI3	I will interact with others on social media	
Perceived Ease of Use (PEOU)	PEOU1	For me, sharing information on social media is not difficult for me, it is an easy thing	Davis (1989), Martín-García et al. (2022), Venkatesh (2000)
	PEOU2	The social media platform’s interactive interface is concise and easy to understand and operate	
	PEOU3	Learning how to comment, retweet and share information on social media is easy for me	
Information Sharing Behavior (ISB)	ISB1	I often share information on social media	Dang (2021)
	ISB2	I often share knowledge and experience on social media	
	ISB3	I am always willing to share information with other users on social media to help them when they make a request or need help	

## 4 Data analysis

In this study, the normality assumption was examined as part of the evaluation of validity and reliability to ensure data quality. Firstly, the distributional characteristics of the data were verified through skewness and kurtosis analyses. Skewness and kurtosis are summary statistics that measure the degree of deviation from normality. Given the robustness of PLS-SEM to non-normal data, values between −2 and 2 are generally considered acceptable (Santha Vaithilingam et al., 2024). In this study, the statistics for all variables fell within the range of −2 to 2, meeting the criteria. Secondly, a visual diagnosis was conducted using Q-Q plots, which revealed that the data points exhibited an approximately symmetric distribution and closely clustered around the theoretical normal curve. Based on a combination of quantitative metrics and graphical inspection results, the data in this study satisfied the normality test standards.

### 4.1 Descriptive statistics

Table 2 shows the basic information of the respondents who participated in this questionnaire survey, among which 59.6% are men and 40.4% are women. Most of the respondents are mainly 55–69 years old, bachelor’s degree or below, married, with an annual family income of more than 50,000 yuan and older adults with friends in life. As far as physical condition is concerned, 63.3% of older adults feel that they are in good health. In addition, more than 90% of the respondents have been exposed to social media such as WeChat, Weibo, Tiktok and REDnote for more than half a year, and more than 80% of older adults use social media almost every day.

**Table 2:** Descriptive statistics of population variables.

Population variables	Property value	Frequency	Proportion (%)
Gender	Male	177	59.6
	Female	120	40.4
Age	55–59 years old	141	47.5
	Aged 60–64	90	30.3
	Aged 65–69	45	15.2
	70–74 years old	12	4.0
	Aged 75 and above	9	3.0
Highest qualification	Primary school and below	20	6.7
	Junior high school	40	13.5
	Senior high school/Secondary technical school/Technical school/vocational high school	60	20.2
	Associate degree	42	14.1
	Bachelor’s degree	113	38.1
	Master’s degree or above	22	7.4
Marital status	Married	267	89.9
	Unmarried	15	5.1
	Divorce	8	2.7
	Widow	7	2.3
Household annual income	Less than 50,000 yuan	40	13.5
	50,000–100,000 yuan	71	23.9
	110,000–200,000 yuan	106	35.7
	Over 200,000 yuan	80	26.9
Physical condition	Health	188	63.3
	Normal	104	35.0
	Poor	5	1.7
Do you have many friends in your life	Yes, and there are many	162	54.5
	Yes, only a few friends	132	44.5
	No	3	1.0
Exposure time to social media such as WeChat/Weibo/Tiktok/REDnote	Less than half a year	16	5.4
	Half a year to 1 year	39	13.1
	1–2 years	63	21.2
	More than 2 years	179	60.3
How often you use social media such as WeChat/Weibo/Tiktok/REDnote	Almost every day	240	80.8
	At least once a week	40	13.5
	At least once a month	13	4.4
	Less than once a month	4	1.3

### 4.2 Measurement model verification

#### 4.2.1 Common method bias test

Aiming at the common method bias problem of sample data, we use Harman single factor method to reduce the dimensionality of factor structure. The results of factor analysis show that the KMO value is 0.875, and the approximate chi-square of Bartlett sphericity test is 3247.925, which indicates that it is suitable for factor analysis. The results of principal component analysis show that a total of 6 common factors with eigenvalues greater than 1 are precipitated, and the cumulative variance interpretation rate of the first common factor is 34.004%, which is lower than the threshold of 40%, indicating that there is no serious common method bias problem in the measurement items of this study.

#### 4.2.2 Reliability and validity test

In this study, Cronbach’s Alpha and Composite Reliability (CR) were used to evaluate the internal consistency of questionnaire measurement items, and Average Variance Extracted (AVE) was used to measure the convergence and effectiveness of questionnaire items. As shown in Table 3, the Cronbach’s Alpha of each latent variable in this study is greater than 0.8, indicating that the scale has good validity. From the convergence validity results, the CR value of each latent variable is greater than 0.7, the AVE value is greater than 0.5, and the standardized factor load of each observed variable is between 0.599 and 0.985, which indicates that the scale has good convergence validity.

**Table 3:** Reliability and convergence validity test results.

Latent variables	Observed variables	Parameter significance estimation				Normalized factor load	Cronbach’s Alpha	CR	AVE
		Unstd.	S. E.	t-value	*p*				
PR	PR1	1	–	–	–	0.766	0.874	0.878	0.592
	PR2	1.136	0.076	14.967	***	0.849			
	PR3	1.121	0.077	14.622	***	0.83			
	PR4	0.68	0.063	10.82	***	0.633			
	PR5	0.712	0.055	13.032	***	0.748			
TA	TA1	1	–	–	–	0.819	0.832	0.837	0.565
	TA2	1.151	0.079	14.631	***	0.83			
	TA3	0.854	0.075	11.409	***	0.657			
	TA4	0.87	0.073	11.947	***	0.684			
PE	PE1	1	–	–	–	0.805	0.838	0.843	0.642
	PE2	1.177	0.089	13.23	***	0.779			
	PE3	1.139	0.083	13.69	***	0.82			
SOI	SOI1	1	–	–	–	0.794	0.817	0.819	0.602
	SOI2	0.974	0.081	12.008	***	0.745			
	SOI3	0.963	0.077	12.462	***	0.788			
PEOU	PEOU1	1	–	–	–	0.762	0.830	0.831	0.621
	PEOU2	0.98	0.078	12.483	***	0.767			
	PEOU3	1.155	0.088	13.197	***	0.833			
ISB	ISB1	1	–	–	–	0.846	0.873	0.873	0.696
	ISB2	1.066	0.061	17.401	***	0.869			
	ISB3	0.857	0.056	15.381	***	0.786			

^***^p < 0.001.

Table 4 lists the correlation coefficients of six latent variables and the square root of the AVE value of each latent variable. Among them, the square root value of AVE of each latent variable is greater than the absolute value of its correlation coefficient with other variables, indicating that the scale has good discriminative validity.

**Table 4:** Results of discriminative validity test.

Latent variables	ISB	PEOU	SOI	PE	TA	PR
ISB	**0.834**					
PEOU	0.619**	**0.788**				
SOI	0.492**	0.488**	**0.776**			
PE	0.473**	0.335**	0.422**	**0.801**		
TA	−0.558**	−0.392**	−0.279**	−0.291**	**0.752**	
PR	0.508**	0.44**	0.263**	0.201**	−0.483**	**0.769**

^**^p < 0.01, and the bold number on the diagonal line is the square root of the AVE value of the corresponding latent variable.

### 4.3 Hypothesis testing

**Figure 2:**
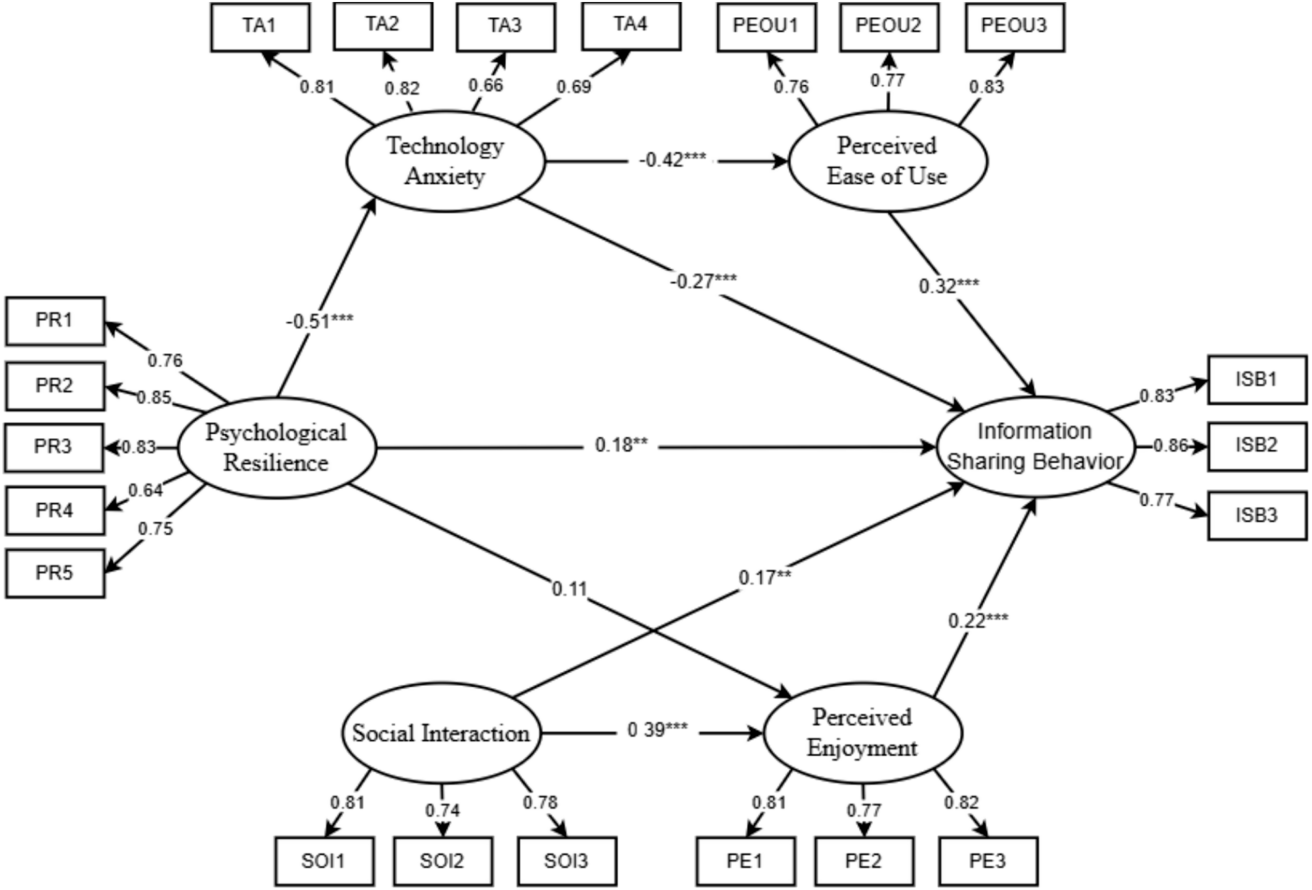
Model with results from SEM.

This study employs structural equation modeling (SEM) to examine the hypothesized relationships among variables, with all analyses conducted using AMOS 28.0 software. Specifically, AMOS 28.0 was utilized to test the proposed model and analyze the path coefficients between variables, thereby determining their interrelationships and validating the hypothesis results. The model fitting results are shown in Table 5, and all kinds of fit index meet the standards, so the model fits well. The hypothesis test results are shown in Table 6 and Figure 2, except for the path “PR → PE ( β = 0.108, *p* = 0.103 > 0.05) “, the significance probability values P of other paths are significant (*p* < 0.05), among which the standardization coefficients of paths “PR → ISB,” “PEOU → SOI,” “SOI → ISB,” “SOI → PE” and “PE → ISB” are greater than 0, and the paths are positively correlated. The normalization coefficients of the paths “PR → TA,” “TA → PEOU” and “TA → ISB” are less than 0, and the paths all show significant negative effects. From the above analysis, it can be seen that the assumption H2 is not supported, but H1 and H3 - H9 are supported.

**Table 5:** Model fitness test results.

Fit index	CIN/DF	RESEA	GFI	NFI	IFI	CFI	PGFI
Standard	<2	<0.05	>0.9	>0.9	>0.9	>0.9	>0.5
Result value	1.579	0.044	0.921	0.918	0.968	0.968	0.694
Model fitting judgment	Yes	Yes	Yes	Yes	Yes	Yes	Yes

**Table 6:** Hypothesis testing.

Hypotheses	Path	Normalization coefficient β	S. E.	C. R.	P	Test results
H1	PR → TA	−0.508	0.066	−7.485	***	Support
H2	PR → PE	0.108	0.057	1.630	0.103	Not supported
H3	PR → ISB	0.184	0.068	2.856	0.004	Support
H4	TA → PEOU	−0.418	0.063	−5.963	***	Support
H5	TA → ISB	−0.269	0.076	−3.835	***	Support
H7	PEOU → ISB	0.324	0.075	5.182	***	Support
H8	SOI → ISB	0.169	0.073	2.722	0.006	Support
H9	SOI → PE	0.390	0.071	5.353	***	Support
H10	PE → ISB	0.220	0.073	3.638	***	Support

***p < 0.001.

### 4.4 Mediation effect test

In this study, the Bootstrap intermediary test method is run by AMOS 28.0 software, and the samples are taken repeatedly for 5,000 times, and the upper and lower confidence intervals of Percentile indirect effects with 95% confidence are obtained. As shown in Table 7, the estimated value of PR indirectly affecting ISB through TA is 0.137, and it passed the significance test (*p* < 0.05); The estimated value of SOI indirectly affecting ISB via PE was 0.086, and it passed the significance test (*p* < 0.05). At 95% percentile confidence level, its confidence intervals for indirect effects were [0.041, 0.233] and [0.027, 0.158] and for direct effects were [0.045, 0.323] and [0.018, 0.318]. In the above intervals, 0 was not included, indicating that the mediating effects were significant and both TA and PE played partial mediating roles. This suggests that older adults’ psychological resilience influences their information-sharing behavior by moderating technical anxiety, while perceived enjoyment acts as a partial mediator between social interaction and information-sharing behavior. Thus, Hypotheses H6 and H11 are supported.

**Table 7:** Results of mediation effect test.

Effect type	β	Percentile 95% CI		*p*
		Lower	Upper	
The Direct Effect of PR on ISB	0.184	0.045	0.323	0.009
Indirect effects of PR _ TA _ ISB	0.137	0.041	0.233	0.006
The total effect of PR on ISB	0.413	0.289	0.536	***
Direct Effects of SOI on ISB	0.169	0.018	0.318	0.027
Indirect effects of SOI _ PE _ ISB	0.086	0.027	0.158	0.002
The total effect of SOI on ISB	0.254	0.105	0.406	0.001

^***^p < 0.001.

## 5 Discussion

In the context of rapid digital technological advancement and deepening population aging, the digital divide faced by older adults has become increasingly prominent, making their information-sharing behaviors on social media platforms an important academic research topic. The widespread use of social media not only provides older adults with new avenues for emotional communication, health management, and social participation, but also opens up new possibilities for alleviating loneliness and enhancing life satisfaction and well-being. However, with the decline of physical functions and cognitive abilities, older adults face numerous challenges in adapting to digital technologies. Most current social media platforms have problems such as complex functions and inadequate interface design (e.g., too small fonts), which make older adults highly prone to negative emotions such as technological anxiety and stress during use. These negative emotions not only significantly weaken older adults’ perceived enjoyment and ease of use of the platforms, but also inhibit their information-sharing behaviors.

Based on the Technology Acceptance Model (TAM) and the Uses and Gratifications Theory (UGT), this study systematically explores the complex relationships among psychological resilience, technology anxiety, perceived enjoyment, perceived ease of use, social interaction, and information-sharing behaviors of older adults. Empirical findings show that psychological resilience, social interaction, perceived enjoyment, and perceived ease of use have significant positive effects on older adults’ information-sharing behaviors; technology anxiety significantly inhibits information-sharing behaviors and reduces older adults’ perceived ease of use of social media. Further analysis reveals that psychological resilience promotes information-sharing behaviors through direct effects and an indirect pathway of reducing technology anxiety, but there is no significant direct association between psychological resilience and perceived enjoyment. In addition, social interaction indirectly promotes older adults’ information-sharing behaviors through the mediating effect of perceived enjoyment. This study reveals the collaborative mechanism of multiple factors, providing important theoretical foundations for promoting age-friendly transformation of social media and improving digital literacy among older adults.

### 5.1 Theoretical implications

This study focuses on the social media context, and deeply explores the structural associations among psychological resilience, technology anxiety, social interaction, perceived enjoyment, perceived ease of use, and information sharing behavior of older adults. The results show that: firstly, the psychological resilience, social interaction, perceived enjoyment and perceived ease of use of older adults all play a positive role in promoting their information sharing behavior; On the contrary, technological anxiety has a significant negative inhibitory effect on the information sharing behavior of older adults. The above research conclusions are consistent with the research results of scholars such as Martel and Alena on various technology use behaviors such as false news sharing behavior, social networking site use, and technology adoption (Bermes, 2021; Dabbous and Aoun Barakat, 2023; Ha and Park, 2020; Kavandi and Jaana, 2020; Lee and Ma, 2012; Martel et al., 2020; Ramírez-Correa et al., 2019). Secondly, psychological resilience presents a dual path in influencing the information sharing behavior of older adults. Directly, psychological resilience influences the information sharing behavior of older adults; indirectly, it exerts influence through affecting the technology anxiety of older adults. Third, technology anxiety has a direct impact on the perceived ease of use of social media in older adults. When older adults develop technology anxiety, it significantly reduces their perceived ease of use of social media. This finding is consistent with the results that technology anxiety negatively affects perceived ease of use, as confirmed by Antonio’s research investigating the willingness to use technology in older adults (Martín-García et al., 2022). Interestingly, and contrary to initial expectations, the analysis revealed that there is no significant direct association between the level of psychological resilience and perceived enjoyment of older adults. However, social interaction plays an important role in affecting perceived enjoyment. It can not only directly and positively affect perceived enjoyment, but also indirectly promote the information sharing behavior of older adults through the mediation variable of perceived enjoyment. This mediating effect is consistent with Rauwers’ research finding that interactivity can affect users’ attitudes toward e-magazines through the mediating variable of perceived enjoyment (Rauwers et al., 2019).

The lower level of psychological resilience in older adults may lead to increased feelings of technology anxiety, which may affect their information behavior. The low level of psychological resilience of older adults means that their self-adjustment and recovery ability in the face of stress and frustration is weak. Older adults are often considered vulnerable because of declining cognitive level, memory and physical fitness (Ghasemaghaei et al., 2019). In the social media environment, when encountering technical-related problems such as operating complex and unfamiliar software functions and frequent system updates, insufficient technical ability and fear of powerlessness often cause nervousness, uncertainty, fear and other anxious states when using technology for older adults (Cheng et al., 2023; Troisi et al., 2022). When older adults lack sufficient psychological resilience to cope with these challenges, it is often difficult for them to effectively alleviate their inner tension and anxiety, which may lead to the aggravation of technology anxiety. In terms of emotional regulation, such elderly people lack an effective emotional buffering mechanism. Once they encounter frustration caused by technical problems, negative emotions will accumulate rapidly, and it is difficult to relieve anxiety through self-appeasement and positive attribution like individuals with high psychological elasticity. From the perspective of internal mechanism, psychological resilience, as the key psychological trait of individuals to cope with stress, has a profound impact on the way older adults deal with technical problems. At the cognitive level, older adults with low psychological resilience may be more inclined to regard technical problems (such as information sharing and information search) as insurmountable obstacles, focusing too much on their own lack of ability, thus amplifying the difficulty of the problem and causing anxiety, which makes them often take an evasive attitude toward technology.

Contrary to initial expectations rooted in UGT, this study found no significant link between psychological resilience and perceived enjoyment. This suggests that, in older adults, emotional gratification from social media may be influenced more by interface usability and content relevance than by internal coping traits such as resilience. The lack of correlation between resilience and enjoyment is theoretically significant and this non-significant association challenges the prevailing assumption in UGT that psychological resilience consistently enhances enjoyment across contexts. In previous studies, individuals with high psychological resilience are generally believed to have a better sense of pleasure from various activities (Jo and Baek, 2023), but this relationship does not hold in studies targeting older adults in social media settings. This may be because the factors that affect the perceived enjoyment of older adults on social media are extremely complex, such as perceived ease of use, perceived informativeness (Holdack et al., 2022), etc. Moreover, the measurement items for perceived enjoyment may not fully capture the subjective experiences of older adults. In addition, the physical abilities and cognitive behaviors of older people often become the main barriers to the use of apps by older people (Wildenbos et al., 2018), the design and functions of social media platforms are mixed, which has a significant impact on the experience of older adults. The possibility of related functions providing users with actions establishes an intuitive mapping relationship between the objective information environment and the consciousness of users (Zhao et al., 2013). If the platform interface design is unfriendly and the operation is complicated, even older adults with high psychological resilience will be difficult to enjoy it. In addition, the fit between older adults’ own interests and social media content, and the quality of social feedback they receive on the platform (Rauwers et al., 2019), etc., may have a more direct and significant impact on perceived enjoyment, and these factors to some extent mask the possible positive correlation between psychological resilience and perceived enjoyment.

Current research has insufficiently explored the underlying mechanisms of psychological resilience in the context of digital technology use, particularly lacking joint analyses on its interactions with variables such as technology anxiety and perceived enjoyment. This study innovatively reveals that psychological resilience not only promotes information-sharing behavior among older adults through a direct pathway but also exerts its influence through an indirect pathway by reducing technology anxiety. Meanwhile, it confirms that there is no significant direct association between psychological resilience and perceived enjoyment, thereby providing a new direction for exploring the relationship between individual psychological traits and technology use behaviors. Furthermore, our research breaks away from the traditional research paradigm that focuses solely on the direct impact of single variables. It systematically verifies the mediating roles of perceived enjoyment between social interaction and information-sharing behavior, as well as of technology anxiety between psychological resilience and information-sharing behavior, thereby deepening the understanding of the complex mechanisms underlying information-sharing behavior. Notably, by dissecting the interactive mechanisms among multi-dimensional variables encompassing psychological, technological, and social aspects, the theoretical model constructed in this study holds universal value applicable across different groups and scenarios.

### 5.2 Practical implications

This research highlights the crucial role of older adults’ psychological resilience to cope with technological challenges, which provides a direction for governments, communities, enterprises and related organizations to carry out digital services and education for older adults. Governments and communities can enhance the psychological resilience of older adults by organizing psychological adjustment training courses and holding digital technology experience activities, help them overcome their fear and anxiety about digital technology, and improve their ability to use technology. At the same time, in view of the positive impact of social interaction on the information sharing behavior of older adults, the government and enterprises can also build social platforms for older adults, organize online and offline communication activities, promote the interaction among older adults, and enhance their participation and sense of gain in digital social interaction, thus improving the quality of life in later years. In addition, the research results clarify the inhibitory effect of technology anxiety on the information sharing behavior of older adults and the promotion effect of perceived ease of use, which provides a direct basis for the aging-friendly transformation of social media platforms. Based on the research results, platform developers can optimize the interface design, simplify the operation process, and lower the technical threshold, such as adapting the interface with simplified modes, accessible visual elements, and reduced operational complexity, so as to reduce the anxiety caused by the complex operation of older adults, enhance their perception of the ease of use of the platform, stimulate their enthusiasm for sharing information on the platform, and enrich the digital social ecology of older adults. For example, The “Care Mode” provided by WeChat incorporates design features such as larger font sizes, enhanced color contrast, oversized buttons, and one-tap text-to-speech playback for messages.

### 5.3 Limitations and future research

Focusing on the context of social media, this study systematically analyzes the characteristics of technology anxiety, perceived enjoyment, perceived ease of use and social interaction of older adults when sharing information on social media through the fusion of TAM and UGT, and systematically analyzes the characteristics of technology anxiety, perceived enjoyment, perceived ease of use and social interaction of older adults when sharing information on social media. At the same time, this study also has some limitations. First of all, this study adopts a cross-sectional study design, which lacks longitudinal research. The social media environment is constantly changing dynamically, and the psychological state, technology use ability and information sharing behavior of older adults will also change over time. Through longitudinal research, we can more clearly observe how older adults’ psychological resilience, technology anxiety and other factors dynamically evolve at different time points, and how these changes affect their information sharing behavior. In addition, this study only focuses on Chinese elderly groups, and does not include foreign elderly groups. Different countries have different cultures, social environments and technology popularization, which may affect the information sharing behavior of older adults. Therefore, future research can further explore the influence of cultural and social environment on the information sharing behavior of older adults through cross-cultural comparison. At the same time, combined with the longitudinal research design, in-depth exploration of the causal relationship among various factors and their changing trends over time will help to more comprehensively and deeply reveal the internal mechanism of information sharing behavior of older adults in social media situations.

## Data Availability

The original contributions presented in the study are included in the article/supplementary material, further inquiries can be directed to the corresponding authors.
